# Colorectal cancer risk in East Asian patients with *Helicobacter pylori* infection: A systematic review and meta-analysis

**DOI:** 10.1097/MD.0000000000033177

**Published:** 2023-03-10

**Authors:** Lijuan Ma, Wentao Guo, Zhihui Zeng, Fei Yang, Shufang Tang, Yarui Ling

**Affiliations:** a Shenzhen Traditional Chinese Medicine Anorectal Hospital Futian, Shenzhen, China.

**Keywords:** colorectal cancer, East Asian, *Helicobacter pylori*, meta-analysis, risk

## Abstract

The aim of this systematic review and meta-analysis was to assess the relationship between *H pylori* infection and colorectal cancer risk in East Asian patients.

**Methods:**

Two researchers independently searched for relevant studies in the PubMed, Cochrane, and Embase databases from inception up to April 2022. A meta-analysis was then performed to calculate pooled odds ratios (ORs) with corresponding 95% confidence intervals (CIs) using a random effects model.

**Results:**

Nine studies involving 6355 patients were included. Overall, we observed that *H pylori* infection was associated with an increased risk of colorectal cancer in East Asian patients (OR = 1.48, 95% CI: 1.10–1.99, *I*^2^ = 70%), although significant heterogeneity was identified among studies. Subgroup analysis revealed that *H pylori* infection was associated with an increased risk of colorectal cancer in China (OR = 1.58, 95% CI 1.05–2.37, *I*^2^ = 81%) but not in Japan and Korea (OR = 1.26, 95% CI 0.93–1.70, *I*^2^ = 0%).

**Conclusion:**

This meta-analysis identified a positive association between *H pylori* infection and colorectal cancer risk in East Asian patients, especially in China.

## 1. Introduction

Up to a few decades ago, colorectal cancer was rarely diagnosed; however, it is currently the 4th deadliest cancer worldwide, causing nearly 900,000 deaths every year.^[1]^ In addition to aging populations and unhealthy dietary habits in some high-income countries, adverse risk factors, such as obesity, lack of physical exercise, and smoking, increase the risk of colorectal cancer.^[1]^ Moreover, associations between *H pylori* infection and colorectal cancer have been described.^[2–4]^

*H pylori* infection is a well-known risk factor for various gastrointestinal diseases, such as peptic ulcers and gastric cancer,^[5,6]^ and it is also associated with extra gastric diseases. However, conflicting results on correlations between *H pylori* infection and colorectal cancer have been obtained. Several studies have reported that the proportion of colorectal cancer patients with *H pylori* infection is significantly higher than that of patients with no infection,^[7,8]^ whereas other studies have disputed this.^[9]^ Thus, the biological basis of associations between *H pylori* infection and colorectal cancer remains uncertain. One possibility is elevated serum gastrin levels in patients with *H pylori* infection.^[10]^ Gastrin receptors have been identified on various colon cancer cell lines, and endogenous serum gastrin levels have been reported to be correlated with colonic neoplasm risk.^[11]^

Approximately half of the world’s population is infected with *H Pylori*.^[12]^ Also, colorectal cancer incidence rates and *H pylori* infection rates in East Asia are high. In China, the colorectal cancer burden is rapidly increasing; in 2020, 555,000 new colorectal cancer cases were identified, which accounted for 2.9% of the global cancer burden.^[13]^ *H pylori* infection rates in China were reported to be 41.5% to 72.3%.^[14]^

Meta-analyses are used to summarize data from multiple studies and provide accurate estimates of biological issues. Therefore, we conducted a systematic review and meta-analysis using published data on East Asian populations to evaluate relationships between *H pylori* infection and colorectal cancer risk. Our research may contribute to a more comprehensive understanding of colorectal cancer risk in patients with *H pylori* infection.

## 2. Data sources and searches

Two researchers (LJ-M and YR-L) independently searched the PubMed, Cochrane, and Embase databases from inception to April 2022 using the following keywords: “*H pylori*,” “*H pylori* infection,” “Colorectal cancer,” “Colorectal carcinoma,” and “Colorectal tumor.” We also searched the reference list of all obtained studies to avoid missing any studies. Titles and abstracts were first independently screened by 2 researchers (LJ-M and SF-T). Then, the remaining studies were reviewed and identified according to the inclusion criteria. Differences between the opinions of 2 researchers (LJ-M and SF-T) were resolved through discussion. Ethics approval was not required as no patient data were accessed.

## 3. Inclusion and exclusion criteria

### 3.1.1. Inclusion criteria.

1. Studies investigating relationships between *H pylori* infection and colorectal cancer.
2. Contact *H pylori* infection confirmed using invasive (endoscopy, biopsy, and histopathology) and noninvasive tests (e.g., 13C urea breath test, immunoglobulin G detection, polymerase chain reaction, and fecal antigen); and.
3. Histological examinations confirming colorectal cancer.

### 3.1.2. Exclusion criteria.

1. No population research (cell line and animal research).
2. Abstracts, letters, reviews, or other non-research articles; and.
3. non-English full-text articles.

## 4. Data extraction

Two researchers (ZH-Z and F-Y) extracted the following information using predesigned data extraction guides: publication year, country, author, sample size, colorectal cancer cases, control cases, *H pylori* infection cases, and non-*H pylori* infection cases.

## 5. Evaluation of quality data synthesis and analysis

We used the Newcastle Ottawa scale (NOS) to assess the methodological quality of the included studies.^[15]^ NOS includes 3 categories (selection, comparability, and results) and 8 items. The NOS score range is 0 to 9 stars. If the total score is ≥ 6, the research is of high quality; if the score is 3 to 5, the research is of medium quality; and if the score is < 3, study quality is low and is excluded. Evaluations were conducted by 2 researchers independently and differences were resolved through discussion.

## 6. Statistical analysis

Data were analyzed in Revman 5.3. Correlations between *H pylori* infection and colorectal cancer risk were calculated using odds ratios (ORs) and corresponding 95% confidence intervals (95% CIs). Both *I*^2^ and X^2^ statistics were used to test heterogeneity across studies; significant heterogeneity was defined as *I*^2^ > 50%. A random effects model was used when significant heterogeneity was identified; otherwise, a fixed effects model was used. Probable publication bias was estimated by constructing a funnel plot. *P* values < .05 were considered statistically significant.

## 7. Study selection and characteristics

**Figure 1.**
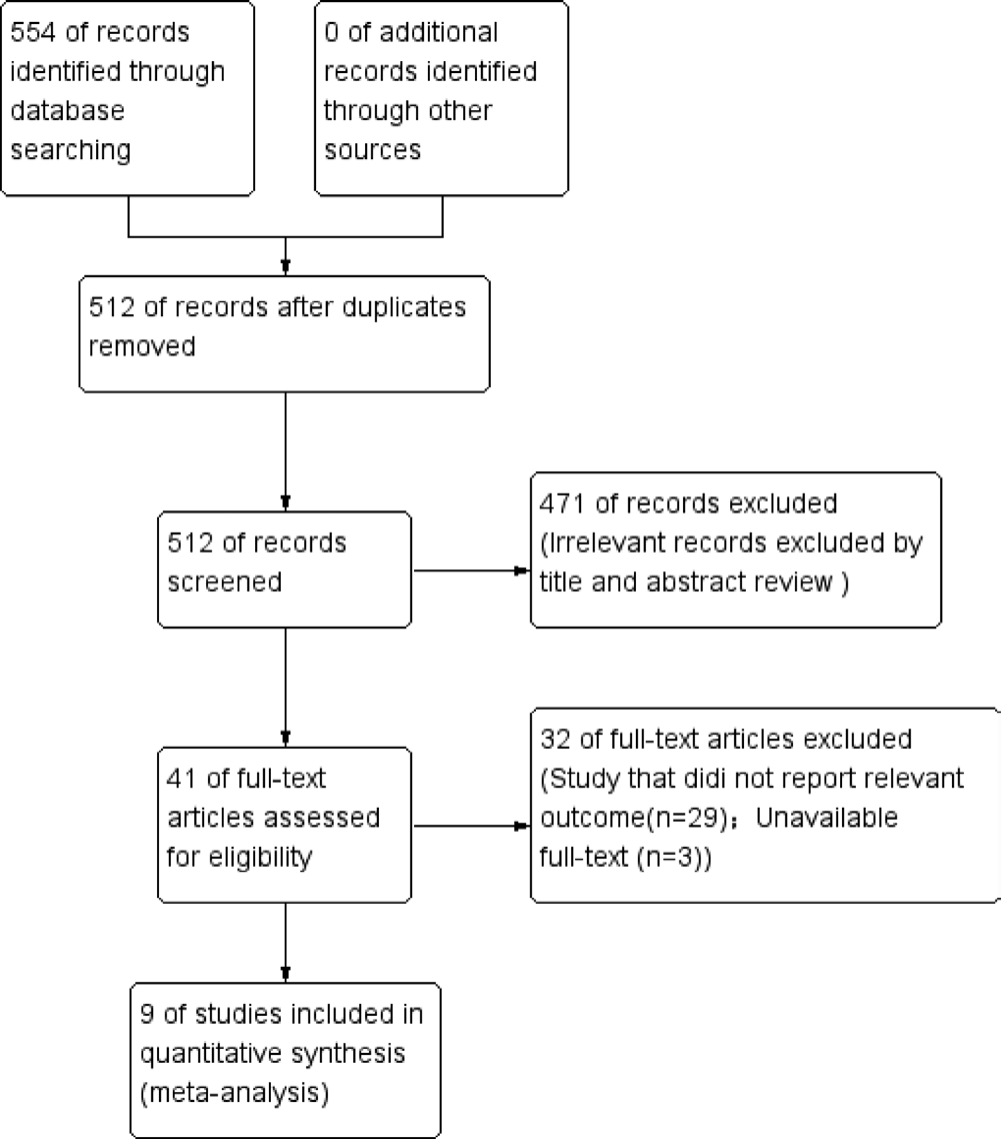
Flow chart showing study selection.

A study selection flow chart is shown (Fig. 1). In total, 554 studies were identified from literature searches, of which, 42 duplicates were discarded. Based on titles and abstracts, 471 studies were excluded. After a full-text review of the remaining 41 studies, 32 were excluded because they did not report relevant results (n = 29) and full texts could not be obtained (n = 3).

Finally, the meta-analysis included 9 studies (3 cross-sectional and 6 case-control studies) involving 6355 patients.^[16–24]^ These studies were published between 2003 and 2020. The characteristics of the included studies and NOS quality scores (ranging from 4 to 9) are shown in Table 1. Of the 9 included studies, 6 (66.7%) were rated as high quality.

**Table 1:** Major characteristics of the eligible studies.

Author (Ref)	Year	Country	No.of patients	Study design	Testing for HP infection stage	Study population	NOS score
Wang et al^[16]^	2003	China	387	Case-control	IgG anti-HP antibody	Hospital-based	4
Fujimori et al^[17]^	2005	Japan	669	Cross-sectional	UBT, RUT, or histological examination	Hospital-based	8
Mizuno et al^[18]^	2005	Japan	307	Cross-sectional	IgG anti-HP antibody	Hospital-based	6
Machida-Montani et al^[19]^	2007	Japan	339	Case-control	IgG anti-HP antibody	Hospital-based	8
Wu et al^[20]^	2009	China	635	Case-control	IgG anti-HP antibody	Hospital-based	8
Nam et al^[21]^	2013	Korea	597	Cross-sectional	IgG anti-HP antibody	Community-based	9
Qing et al^[22]^	2016	China	120	Case-control	RUT, or histological examination	Hospital-based	5
Luan et al^[23]^	2019	China	635	Case-control	IgG anti-HP antibody	Hospital-based	5
Wang et al^[24]^	2020	China	2666	Case-control	UBT	Hospital-based	6

HP = *Helicobacter pylori*, IgG = immunoglobulin G, NOS = Newcastle Ottawa scale, RUT = rapid urease test, UBT = urea breath test.

## 8. Colorectal cancer risk

**Figure 2.**
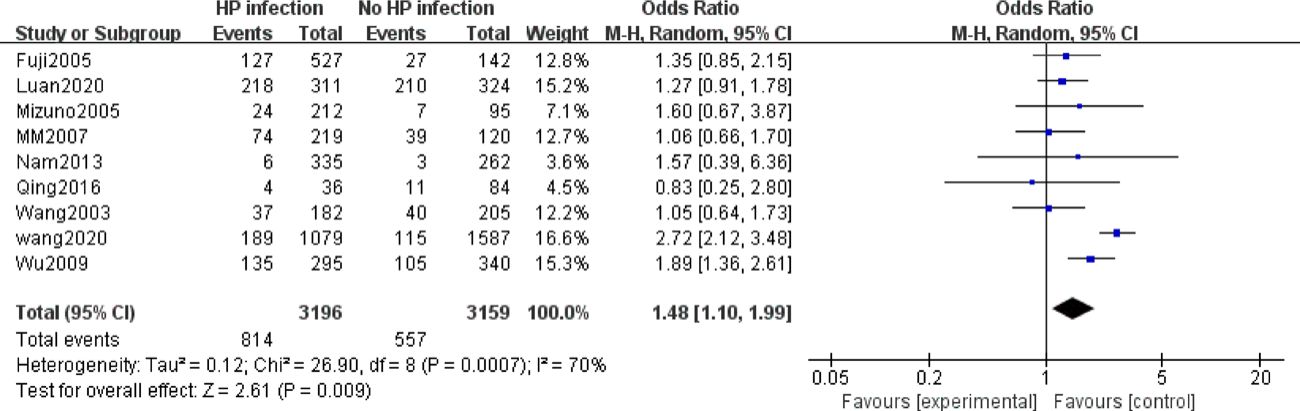
Forest plot of the risk of colorectal cancer with *H pylori* infection versus non- *H pylori* groups.

Figure 2 shows the risk of colorectal cancer in patients with *H pylori* infection. The meta-analysis results showed a significant correlation between *H pylori* infection and colorectal cancer risk in East Asian patients (pooled OR = 1.48, 95% CI: 1.10–1.99, P_heterogeneity_ = 0.0007, *I*^2^ = 70%).

Subgroup analyses were performed by region in selected studies. Studies conducted in China (OR = 1.58, 95% CI: 1.05–2.37) showed a significantly positive association between colorectal cancer risk and *H pylori* infection, whereas those conducted in Japan and Korea did not show this association (OR = 1.26, 95% CI: 0.93–1.70).

## 9. Sensitivity analysis

The results of sensitivity analysis are presented in Table 2. The pooled ORs ranged from 1.05 (95% CI: 1.14–2.12) to 1.46 (95% CI: 1.15–2.13) after 1 study was removed each time, indicating that no individual study substantially influenced the pooled ORs.

**Table 2:** Sensitivity analysis of 1 study was removed each time.

Removed study	OR	95% CI	P_heterogeneity_	*I* ^2^
Wang 2003	1.05	1.14–2.12	0.002	69%
Fuji 2005	1.50	1.07–2.08	0.0005	73%
Mizuno 2005	1.47	1.07–2.02	0.0004	74%
MM 2007	1.56	1.15–2.13	0.002	69%
Wu 2009	1.41	0.99–2.01	0.0004	74%
Nam 2013	1.48	1.08–2.01	0.0003	74%
Qing 2016	1.52	1.13–2.06	0.0006	73%
Luan 2020	1.52	1.09–2.12	0.002	70%
Wang 2020	1.37	1.16–1.63	0.43	0%

CI = confidence interval, OR = odds ratios.

## 10. Publication bias

Following the criteria outlined in the Cochrane Handbook for Systematic Reviews of Interventions, we did not analyze publication bias as no group included > 10 studies.

## 11. Discussion

In this meta-analysis, we identified a significant correlation between *H pylori* infection and colorectal cancer risk in East Asian patients (pooled OR 1.48 [95% CI:1.10–1.99]) in the 2003 to 2020 period. Subgroup analyses by region were conducted on all studies and it was observed that in China, *H pylori* infection was positively correlated with colorectal cancer (pooled OR: 1.58, 95% CI: 1.05–2.37), but in Japan and Korea, *H pylori* infection was negatively correlated with colorectal cancer (pooled OR: 1.26, 95% CI: 0.93–1.70). Sensitivity analysis revealed that our results were reliable and stable; pooled ORs ranged from 1.05 (95% CI: 1.14–2.12) to 1.46 (95% CI: 1.15–2.13) after removal of 1 study each time. The heterogeneity in our analysis may have been caused by differences in study populations and design.

*H pylori* is a recognized human carcinogen and is the main infectious pathogen of single origin in 770,000 cancer cases worldwide every year.^[25]^ Since *H pylori* was identified as a single infectious source in gastric cancer, its putative carcinogenic role has been extended to other malignancies.^[26]^ However, conflicting data exist on the relevance of *H pylori* as a causative agent of colorectal cancer. Indeed, some studies reported a slightly increased risk of colorectal cancer associated with *H pylori* infection, while others disagree with this. We herein identified a moderate association between *H pylori* infection and colorectal cancer risk in East Asian patients.

It is known that *H pylori* infection rates are different between general populations in Western and Eastern countries. Therefore, we analyzed Chinese, Japanese, and Korean subgroups according to regional population differences. *H pylori* infection was positively correlated with colorectal cancer risk in different subgroups, which can be related to the fact that China is a developing country, whereas Japan and Korea are developed countries. In developed countries with advanced economic and medical infrastructure, *H pylori* infections can be detected and controlled at early stages.

The present study had several limitations. We included studies from China, Japan, and Korea, but only 9 studies and 6355 patients were identified, which might be less representative of the corresponding areas. In the future, well-designed studies must be conducted to investigate the prevalence of *H pylori* infection in these areas. Furthermore, study heterogeneity was quite high, which may have led to inaccurate estimates of overall or subgroup prevalence. The sources of heterogeneity may be different populations, areas, time periods, and diagnostic methods. Finally, we only focused on the prevalence of *H pylori* infection in East Asia, which might limit the generalizability of our findings to other regions worldwide.

**Figure 3.**
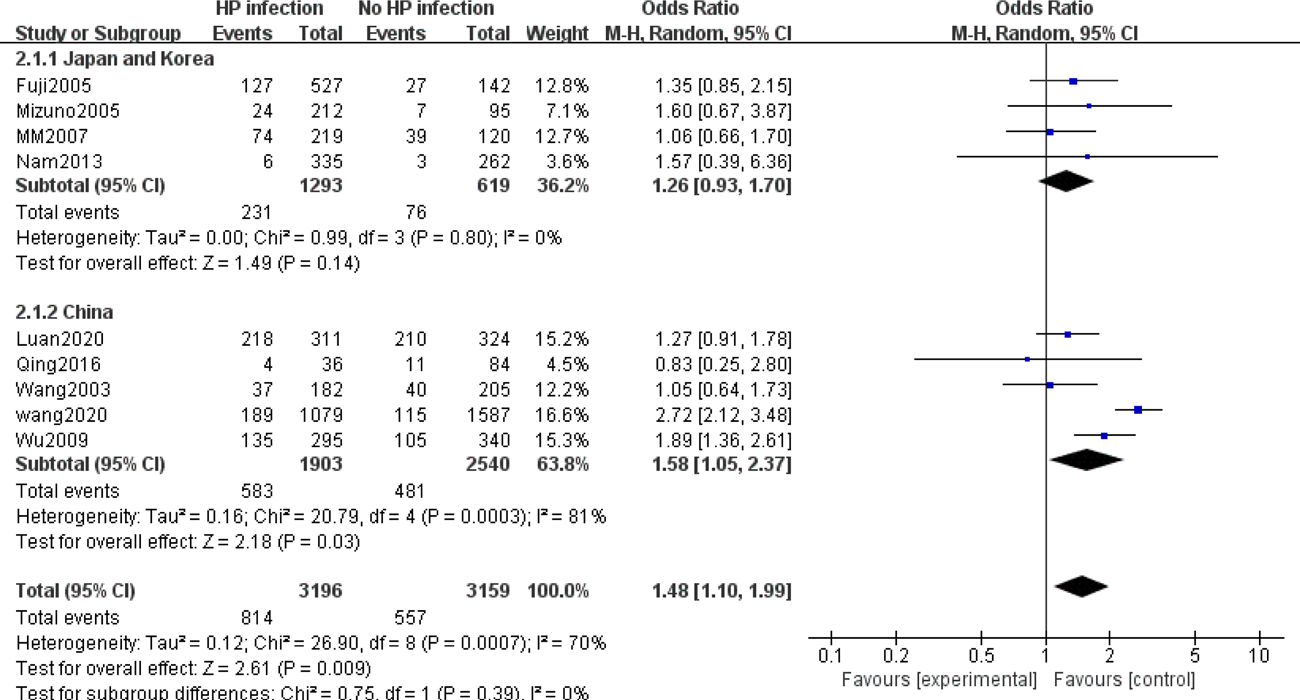
Forest plot of the risk of colorectal cancer with *H pylori* infection versus non- *H pylori* groups with sub-analysis.

## Acknowledgements

The authors thank all study patients and clinical investigators included in this meta-analysis.

## Author contributions

**Conceptualization:** lijuan Ma, Wentao Guo.

**Data curation:** lijuan Ma, Zhihui Zeng, Fei Yang, Shufang Tang, Yarui Ling.

**Formal analysis:** lijuan Ma.

**Software:** lijuan Ma, Yarui Ling.

**Writing – original draft:** lijuan Ma.

**Writing – review & editing:** lijuan Ma.
